# Case Report: A case of primary pericardial mesothelioma treated with multimodal combined therapy

**DOI:** 10.3389/fcvm.2024.1433668

**Published:** 2024-10-07

**Authors:** Jinlan Gong, Xiaofeng Wu, Jiehua Wang

**Affiliations:** Department of Radiation Oncology, Shidong Hospital, Yangpu District, Shanghai, China

**Keywords:** primary pericardial mesothelioma, multimodal therapy, chemotherapy, radiotherapy, immunotherapy, chest pain

## Abstract

**Background:**

Primary pericardial mesothelioma (PMPM) is a rare, aggressive, and lethal form of cancer. Due to its rarity, low incidence and poor prognosis, PMPM has no accepted standard-of-care treatment options with management and outcomes often extrapolated from diffuse pleural mesothelioma. Disease-specific studies are needed to better define PMPM. We report a case of PMPM highlighting the potential role for multimodal combined therapy.

**Case report:**

The patient is a 62 years old female who had nonspecific syndromes and inconclusive image findings in May 2023. Then monthly follow-up echocardiography was performed. Two months later, cardiac ultrasound showed pericardial fluid. Pericardiocentesis with pericardial drain was performed. The fluid was bloody, cultivations for tuberculosis were negative and cytological analysis of the fluid showed no malignant cells. Positron emission tomography-computed tomography revealed that the lesion was localized at the anterior and left part of the mediastinum without distant metastasis. Followed up a pericardiectomy was operated. The diagnosis of PMPM is determined by pathological and immunohistochemical evaluation of tissue specimens. Postoperative patient experienced chest pain, right shoulder and upper limb swelling and pain. Pain management and anticoagulant therapy were administered. The patient underwent multimodal therapy consisting of surgical resection, six cycles of chemotherapy (carboplatin plus pemetrexed) in combination with pembrolizumab, and sequential adjuvant intensity-modulated radiation therapy, totaling 50 Gy in 25 fractions, as the first-line treatment, resulting in complete relief of symptoms and satisfactory outcomes with no complications. Presently, the tumor is under local control, with no signs of distant metastasis, and maintenance immunotherapy is scheduled. Continued observation is necessary for monitoring subsequent disease progression.

**Conclusion:**

PMPM represents a distinct disease with no universally accepted treatment options. The case suggests that multimodal treatment may improve outcomes in selected patients with PMPM.

## Introduction

Primary pericardial mesothelioma (PMPM) is a rare, aggressive, and lethal form of cancer. It arises from pericardial mesothelial cells, with an incidence of less than 0.002%. Various risk factors contribute to this disease, such as asbestos exposure, viral infections, radiation exposure, immunodeficiency, and genetics. Clinical signs include constrictive pericarditis, pericardial effusion, and heart failure. Diagnosis relies on histopathological examination. PMPM is clinically rare with a poor prognosis, typically resulting in an average median survival time of 6–10 months. Currently, treatment lacks prospective evidence and specific clinical recommendations, relying on treatment experience documented in case reports. This report presents a case of PMPM in an adult female with atypical symptoms and explores the potential of multimodal combined therapy as a new treatment option.

## Case report

A 62-year-old female patient presented to our department in September 2023. The patient complained that she suffered from chest pain, right upper limb swelling and fatigue about one week. She was in the state of anxiety and depression for the last 8 years. She was a smoker consuming 1 pack of cigarettes every 4 days. She had no history of occupational asbestos exposure.

**Figure 1:**
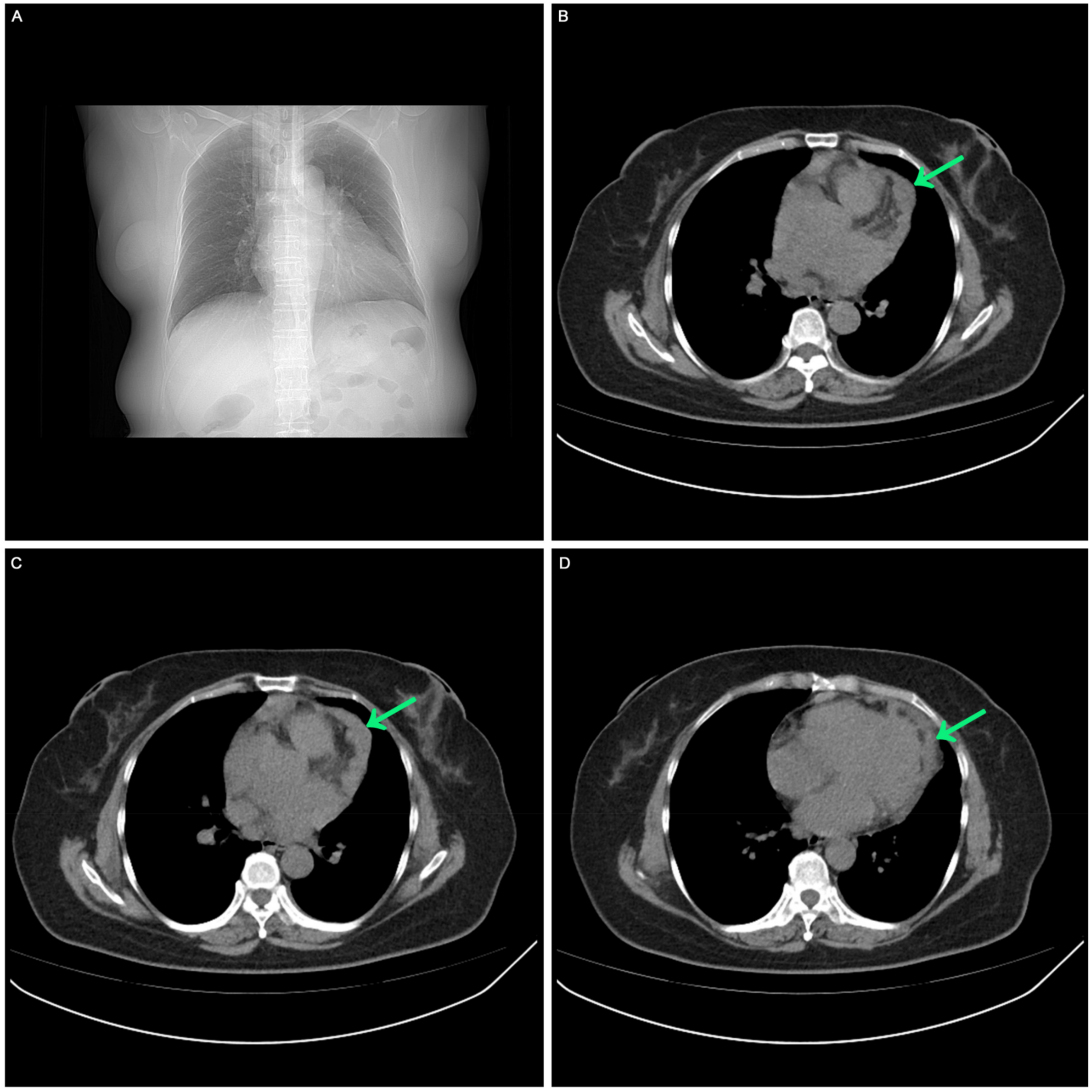
The first visit in May 2023. **(A)** Chest CT scan showed no pleural effusion, no pericardial effusion. **(B, C, D)** Chest CT scan showed irregular low-density mass in the left side of the heart.

A comprehensive medical history was conducted. In May 2023, the patient sought care at our hospital's fever clinic due to “lower fever and dry cough.” Initially, a “respiratory tract infection” was suspected, and chest CT scan was performed and revealed irregular thickened pericardium (Figure 1).

Monthly follow-up echocardiography was performed at a tertiary hospital. Two months later, she suffered from vomiting and fever for several days. Cardiac ultrasound showed pericardial fluid with a width of 2 cm. Pericardiocentesis with pericardial drain was performed. The fluid was bloody, cultivations for tuberculosis were negative and cytological analysis of the fluid showed no malignant cells. Positron emission tomography-computed tomography (PET-CT) was then performed to detect potential metastasis. The result showed 18F-fluorodeoxyglucose (FDG) uptake in the anterior and left part of the mediastinum without distant metastasis. Followed up a pericardiectomy was planned. At operation, partial resection of tumor tissue surrounding the heart and adhesion release of pericardium were performed. The pathology showed a malignant tumor of epithelioid cells. Tumor cells were round or oval in shape, with significant abnormalities. Some areas showed glandular structures, while other areas showed nest-like structures, accompanied by multifocal necrosis. Immunohistochemical analysis confirmed the diagnosis of epithelioid mesothelioma. Immunohistochemical staining results showed the tumor cells expressing WT-1, cytokeratin 5/6, D2-40, CAM5.2, Vimentin, GATA3, while not expressing calretinin, EMA, HBME-1, TTF1. The Ki67 proliferation index was approximately 60%. Six days postoperatively, chest-enhanced CT scan uncovered multiple nodular thickening in the mediastinal anterior and left regions, enlargement of lymph nodes in the 1R low cervical region and right axilla. Additionally, tumor embolus and thrombus formation were observed in the superior vena cava, right brachial vein, right internal jugular vein, and right subclavian vein. Half a month postoperatively, the patient experienced chest pain, right shoulder and upper limb swelling and pain, preventing her from lying flat and necessitating a sitting position. Pain management and anticoagulant therapy were administered at the tertiary hospital. She received oral oxycodone hydrochloride 10 mg every 12 h and rivaroxaban 10 mg once daily. However, as the symptoms persisted, the patient sought further treatment at our hospital in September 2023.

**Figure 2:**
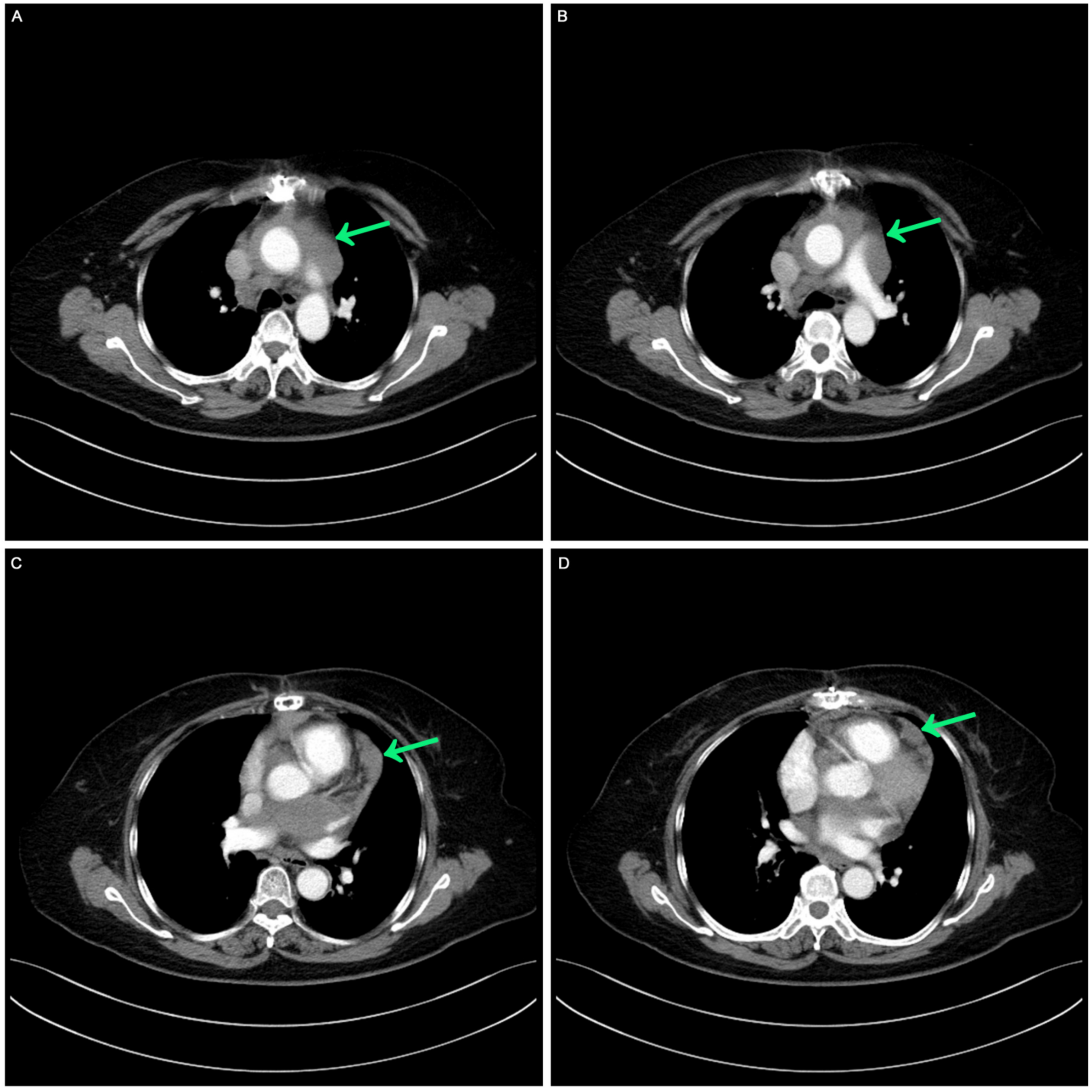
Postoperative contrast-enhanced CT: mild thickening of the pericardium before the third cycle of chemotherapy. **(A,B)** Computed tomography image showed masses around the aorta and pulmonary arteries. **(C,D)** Computed tomography image shows mild thickening of the pericardium.

**Figure 3:**
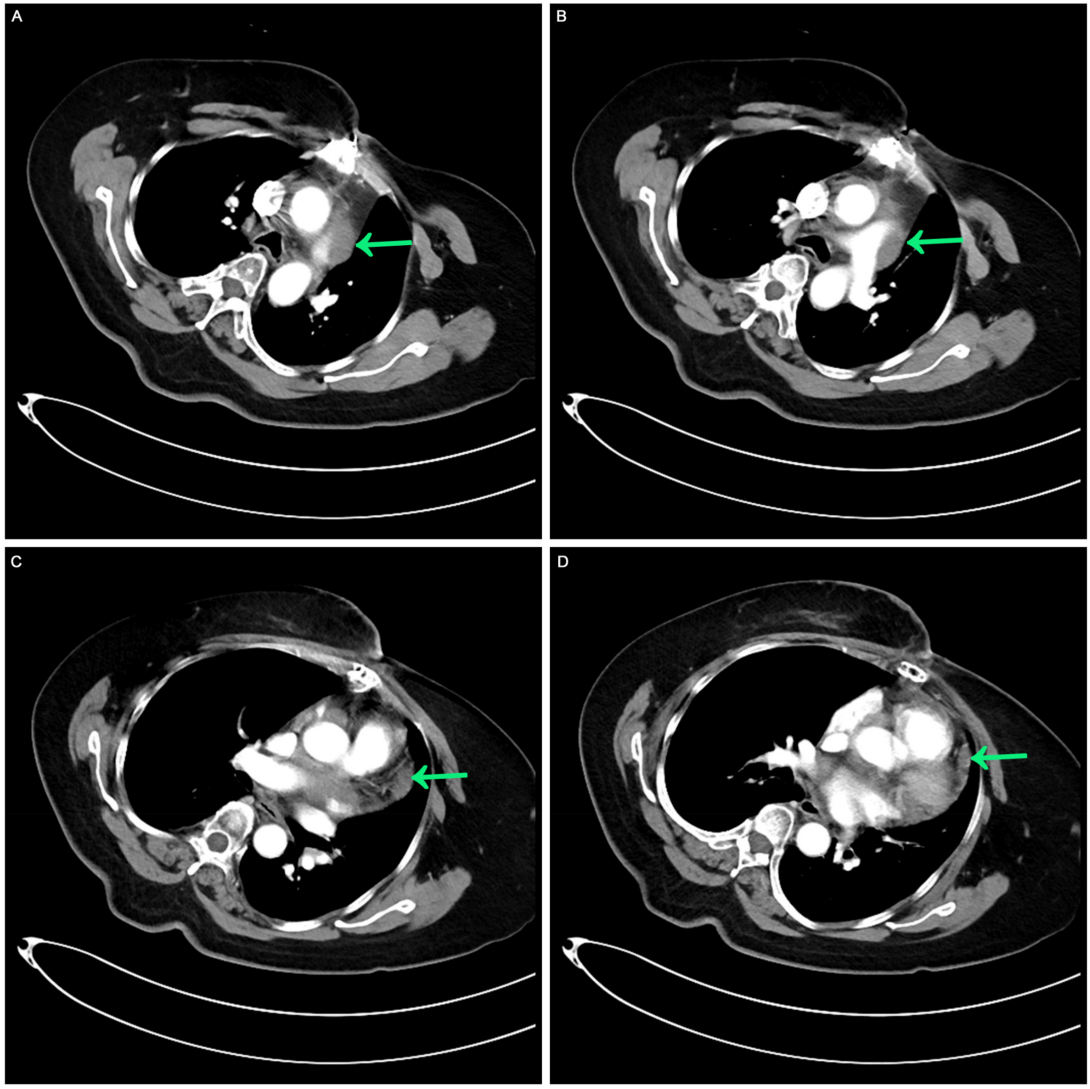
Contrast-enhanced CT after the completion of multimodal treatment. **(A,B)** The masses around the aorta and pulmonary arteries were retracted. **(C,D)** A comparable slight thickening of the pericardium was noted, receding from previous observations.

Before systemic treatment, the patient's body weight and length were 70 kg and 163 cm, respectively, and her blood pressure, heart rate, and respiratory rate were 139/84 mmHg, 106 beats/min, and 20 breaths/min, respectively. The physical examination was notable for swelling of the right limb and jugular vein elevation. The initial laboratory analysis revealed that the white blood cell count was 10,910 cells/mm^3^, the BUN/creatinine ratio was 24.4, the high sensitivity troponin I level (hstroponin I) was <0.02 ng/ml, BNP was 66.9 pg/ml, and blood CA125 level was 53.36 u/ml, non-small cell antigen was 55.32 ng/ml, and NSE was 36.03 ng/ml. Electrocardiography showed sinus tachycardia without abnormal T waves or ST segment changes. Echocardiography revealed a solid hypoechoic area in the pericardial cavity above the left atrium and anterior to the left pulmonary artery, measuring approximately 13 × 3 cm, with an unclear border, irregular shape, and no evident blood flow on color Doppler flow imaging. Due to the patient's inability to lie flat, no chest CT or heart MR signs were obtained before chemotherapy. We discussed the following therapeutic regimen. The treatment plan involved chemotherapy combined with immunotherapy administered from September 07, 2023, to January 19, 2024, comprising 6 cycles. The specific drug regimen consisted of pemetrexed 500 mg/m^2^, carboplatin AUC = 5, and pembrolizumab 200 mg, repeated every 3 weeks. During the third cycle of chemotherapy, sequential pericardial occupying lesion radiotherapy was conducted with a dose of 50Gy/25Fx. Following the first cycle of chemotherapy, cancer pain subsided, leading to the discontinuation of painkillers and enabling the patient to sleep semi-reclined with elevated pillows at night. A reassessment before the third cycle of chemotherapy indicated regression of the pericardial mass (Figure 2). After completing first-line treatment, a follow-up evaluation two months later revealed further regression of the pericardial mass (Figure 3).

After 3 cycles of chemotherapy, CA125, non-small cell-related antigen, and NSE levels returned to normal. Grade II bone marrow suppression, fatigue, nausea, vomiting, and gastrointestinal reactions occurred during systemic treatment, without other treatment-related side effects. After continuing the follow-up process, the patient had no arrhythmia or dyspnea or pain. CT scan revealed lessened pericardial thickening, normal heart size, no pleural or pericardial effusion except mild elevation of the left diaphragm.

**Figure 4:**
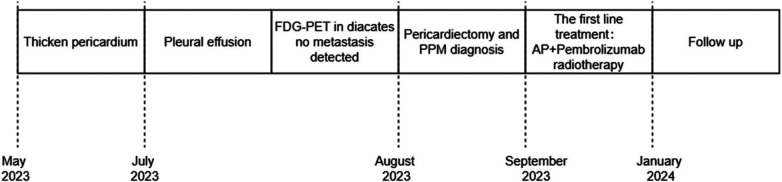
The treatment time nodes.

This case entailed multimodal treatment comprising surgical resection, chemotherapy combined with immunotherapy, and sequential adjuvant pericardial intensity-modulated radiotherapy. Presently, the tumor is under local control, with no signs of distant metastasis, and maintenance immunotherapy is scheduled. Continued observation is necessary for monitoring subsequent disease progression. The treatment time nodes are shown in the Figure 4.

## Discussion

PMPM stands out as an aggressive tumor primarily marked by local infiltration and an unfavorable prognosis. While about 60% of mesotheliomas emerge in the pleura and approximately 35% in the peritoneum, pericardial occurrences are exceptionally rare, making up merely 1% of all mesotheliomas (1, 2). Its detection rate in autopsies of 500,000 individuals ranges from only 0.006% to 0.0022% (1, 2). It can affect individuals across all age groups, with a male predominance and a male-to-female ratio of about 2:1, with the average age of onset hovering around 46 years (3). Given the pericardium's specific location, treating this disease yields poor outcomes, with an average survival time of merely 6–10 months (4).

The clinical manifestations of PMPM are nonspecific and can be confused with tuberculous pericarditis, constrictive pericarditis, coronary heart disease, and cardiomyopathy, leading to a high clinical misdiagnosis rate (5–7). Radiologic and sonographic findings with PMPM can be nonspecific, conclusive diagnostic evaluation often requires tissue sampling. Histopathological diagnosis of pericardial tissue is a powerful diagnostic tool, and immunohistochemistry can enhance accuracy and decrease misdiagnosis rates. Non-invasive examinations play a crucial role in PMPM diagnosis, which remains a diagnosis of exclusion. The disease's etiology is still unclear, and its connection with asbestos exposure is uncertain. In this instance, relevant occupational exposure and a family history of tumors were denied by the patient, and the specific cause of the disease remains unknown. The patient initially had a fever and cough, presenting with atypical early symptoms. Despite close echocardiographic monitoring, it took three months from symptom onset to diagnosis. In many PMPM cases, diagnosis may take several months to over a year, with a median time of six months (8). This delayed recognition is likely due to PMPM's rarity, nonspecific symptoms, similar presentation to benign pericardial diseases, and challenges associated with tissue sampling in this anatomical site. Less than a month after pericardial surgery, the patient experienced symptoms resembling unstable angina, along with extensive thrombosis in the venous system and enlargement of multiple lymph nodes, both intrathoracic and extrathoracic, raising concern for lymphoma. Tumor expressed three mesothelial markers, WT1, D2-40 and CK5/6, confirming the diagnosis of mesothelioma. The patient received one cycle of systemic chemotherapy combined with immunotherapy, resulting in significant improvement in clinical symptoms. The patient benefited from combination therapy.

Currently, the treatment of PMPM typically mirrors that of pleural mesothelioma. Multimodal treatment, including surgery, chemotherapy, and radiotherapy, is a potential standard option for resectable pleural mesothelioma. However, the effectiveness of this approach in PMPM is uncertain, there is a lack of prospective evidence and specific clinical recommendations due to its rarity. Current understanding is primarily based on treatment experience derived from case reports.

A retrospective review of 103 PMPM patients conducted by McGehee et al. (9) indicated that those who underwent multimodal therapy experienced better overall survival rates compared to those treated with single-modality therapy. Specifically, patients who received platinum-based chemotherapy with or without pemetrexed, particularly in combination with both, exhibited improved survival, with an average survival of up to 16 months. Offin et al. (10) reported the findings of a prospective study involving 12 PMPM patients spanning from 2011 to 2022. All patients who underwent triple therapy, including surgical resection, adjuvant chemotherapy, and sequential adjuvant intensity-modulated radiotherapy, achieved long-term disease control. The median overall survival for the entire cohort was 25.9 months, while patients receiving triple therapy had a median overall survival of 70.3 months, significantly exceeding the median survival of 8.2 months for patients treated with surgery combined with chemotherapy alone. These findings suggest that initiating first-line triple therapy may lead to optimal long-term outcomes in specific patient populations.

Recent studies have highlighted the potential of immunotherapy in mesothelioma treatment, although biomarkers predicting efficacy remain uncertain. Presently, there are no reports on first-line chemotherapy combined with immunotherapy for pericardial mesothelioma. However, in the first-line treatment of unresectable pleural mesothelioma, the CheckMate-743 study revealed a median survival of 18.1 months for patients treated with nivolumab + ipilimumab, surpassing the 14.1 months observed in the standard chemotherapy (pemetrexed + cisplatin or carboplatin) group, with greater overall survival benefits noted in non-epithelioid pleural mesothelioma patients (11). The difference was linked to higher PD-L1 expression, correlating with a better response to immune checkpoint inhibitors. It would be valuable to investigate whether this patient's response is PD-L1 dependent, making it important to assess PD-L1 expression through histological analysis. The IND-227 study (12) also demonstrated that patients in the pembrolizumab combined chemotherapy group had a median survival of 17.3 months, with a 3-year survival rate of 25%, compared to 16.1 months and a 3-year survival rate of 17% in the chemotherapy-alone group. Immunotherapy benefits were observed regardless of PD-L1 status. Hence, when evaluating mesothelioma cases, a prompt multidisciplinary team assessment is crucial to identify patients suitable for more aggressive treatment strategies to maximize clinical benefits.

This case employed a more assertive multimodal treatment, including surgical resection, a combination of pemetrexed and carboplatin with pembrolizumab, and pericardial radiation therapy. Presently, the tumor is effectively controlled without cardiac toxic side effects. However, the long-term outcomes and survival benefits require further observation. The report is based on a single case study. Further studies are necessary to validate the effectiveness of the proposed multimodal approach in a broader patient population.

## Data Availability

The original contributions presented in the study are included in the article/Supplementary Material, further inquiries can be directed to the corresponding author.
